# The Vexed Question of Gleditschine

**Published:** 1887-11

**Authors:** 


F. E. DANIEL, M. D., EDITOR AND PUBLISHER

COLLABORATORS:

E. J. Doering, M. D., Chicago.
Geo. Cupples, M. D., San Antonio.
Odo Betz, M. D., Germany.
E. J. Beall, M. D., Fort Worth, Texas.
T. C. Osborn, M. D., Cleburne, Texas.
Wm. Penny, M. D., New York.
H. O. Marcy, M. D., Boston.
C. K. Gregg, M. D., Mexico.
R. M. Swearingen, M. D., Austin.
C. H. Wilkinson, M. D., Galveston.
J. W. McLaughlin, M. D., Austin.
R. H. L. Bibb, M. D., Mexico.

### THE VEXED QUESTION OF GLEDITSCHINE.

The medical world was startled, a few weeks ago, by the announcement of the discovery, by a Mr. Goodman, of wonderful anæsthetic and mydriatic properties in a common, well-known forest tree, indigenious to the South, and the claim was apparently substantiated by reports of experiments by well-known and reliable gentlemen, published in leading MEDICAL JOURNALS, to-wit: Dr. J. H. Claiborne and Dr. H. Knapp, of New York, in the *New York Medical Record* and the *Philadelphia Medical News*.

Just as the profession had begun to rejoice in the discovery, and had accepted the statements of Drs. Claiborne, Knapp, Jackson and others, as conclusive that we had, really, in our midst, available in abundance, an anæsthetic more powerful and much cheaper than cocaine, possessing, in addition, the qualities of atropine, here comes a statement from Messrs. Parke Davis & Co., that they had procured from Lehn & Fink, chemists, New York, *some of the solution with which Drs. Knapp & Claiborne's experiments had been made*, and that upon examination it was found to be a solution of cocaine, and (presumably) atropine; and declaring the whole thing a hoax or a fraud. This turn in the affair left the profession in a state of uncertainty and doubt, the character and standing of Park Davis & Co., entitling their word to belief, while the integrity of Drs. Claiborne, Knapp and Jackson is not for a moment to be impeached. We think we see through the whole matter; both parties are probably partly correct. But how do P. D. & Co. know that the solution sent them was some of the same used by Drs. C. & K?

It seems that Mr. Goodman is a veterinary surgeon, and that he was traveling in East Feliciana, La., where, having occasion to apply a poultice to his horse's leg, he raked up some leaves from the ground for that purpose, without knowing what kind of leaves they were. When he discovered that the poultice produced *anæsthesia* he became interested, and, it seems, "with the simplest kind of domestic utensils," proceeded to make an aqueous extract from some of the same kind of leaves. *This preparation* he put into the hands of Drs. Claiborne & Knapp, as we understand it; and it was with *this preparation* "prepared on the banks of the Mississippi river," that they made their experiments; for, Dr. Claiborne, in an article in *Gaillard's Medical Journal* for November—written, it seems, since doubt has been cast on the subject, says, (page 444) "all the solution of the alkaloid at present possessed, was made before leaving Louisiana; only a few leaves, spines and pods of the tree had been brought to New York."

Now, Lehn & Fink are not mentioned in Dr. Claiborne's article, and just how they got hold of any of "the solution with which Dr. Claiborne and Dr. Knapp's experiments were made," doesn't appear. The probabilities are largely that they did not come in possession of any of *this* solution; but that they may have put up a solution of cocaine and atropine and attempted to palm it off as a solution of the new alkaloid is not only probable, (because Parke Davis & Co. analyzed it and detected the fraud) but is in keeping with the history and record of this enterprising house, for, we have it on the authority of the *Druggists' Circular* that they are now being prosecuted for counterfeiting Merck's labels.

Hence, the fact that Lehn & Fink sold to Parke Davis & Co. (a very foolish thing to do—for more reasons than one) a solution of cocaine under pretense that it was a solution of the new alkaloid *Gleditschine*, does not, in the least, discredit Dr. Claiborne and Dr. Knapp's statements; it does not affect the matter in the least; nevertheless, we are pleased to see Dr. Claiborne and Mr. Goodman are taking steps to verify all that has hertofore been claimed by Dr. Claiborne with regard to the anæsthetic properties of an extract from the leaves of the honey locust. The following letter from Dr. Claiborne to the *New York Medical Record* of November 5, will explain the matter more fully than we could do in the space at our disposal, and is copied *verbatim*:

"DEAR SIR: In view of the recent statements of several analytical chemists that gleditschine, the new local anæsthetic, is nothing but a mixture of cocaine and atropine, I deem that I owe it to the readers of THE MEDICAL RECORD to say a few words on the subject.

"Mr. Goodman has just returned from Bayou Sara, La., where, he states, he has been making the solution of gleditschine. He informs me that he heard for the first time, on his arrival in Bergen Point, N. J., November 1, of the analysis of the chemists, who find the solution to contain atropine and cocaine. He expressed himself as being earnestly desirous of setting himself right in the eyes of the medical profession, and, in order to convince them of the genuineness of his discovery, he agrees to do the following:

"I, Dr. Claiborne, am to select two medical men of this city; they are to select as a committee of two a botanist and a chemist; the botanist is to examine the leaves which will be used, and is to pass an expert opinion as to whether they are the leaves of the *Gleditschia triacanthos*, and if not, of what tree they are leaves. The chemist is to bring the chemicals necessary, these being indicated by Dr. Seward or Mr. Goodman, and is to make the solution from the leaves in possession of Dr. Seward and Mr. Goodman, under their direction. The chemist is then to test the anæsthetic and mydriatic effect of this solution on a human being or lower animal selected by himself (the chemist), and is to state to the world, through the columns of the New York MEDICAL RECORD and the *New York Medical Journal*, whether said solution has anæsthetic and mydriatic effects or not. If cocaine or any mydriatic be employed in the process, to say so; if not, to say not.

"The condition to be imposed is that the chemist, on his word as his bond, is not to divulge the method of procedure of óbtaining the substance.

"This solution, if it be desired, may be placed in the hands of experts, who may pass judgment upon its anæsthetic and mydryatic properties.

"Bergen Point, N. J., is to be the site of the procedure, or, if it be demanded, any other place.

"No one can fail to be struck by the fairness of this proposition.

"Dr. George F. Shrady and Dr. Frank P. Foster have agreed to act as a committee in this matter.

"Mr. Goodman informs me that he has had shipped from New

Orleans a quantity of the leaves of the tear-blanket tree, by one of the Cromwell line of steamers, and that he expects them shortly.

"To my mind the solution seemed to have effects different from cocaine and atropine.

"Moreover, several facts regarding the tree, which have come under my observation, or to which my attention has been called, seemed to warrant the conclusion that the point had not been proven beyond a reasonable doubt.

"In this connection I would say that, with a strong aqueous solution of the gleditschia leaf, obtained through a friend from a chemist in New Jersey, who was experimenting in this direction, I observed in my own eye, and in that of my friend, a dilation of the pupil.

"This lasted in my case for several days. His case was not followed up. No anæsthetic effect was observed in either. Every seeming possibility of error was eliminated. A bottle was used for the solution which had never been used for a mydratic or any other medicine, as far as was known, a new medicine dropper was used, and my own hands were thoroughly washed before commencing, Neither this gentleman nor myself had touched a bottle containing any mydriatic for twenty-four hours. It is pertinent to remark that the dilatation was slight, and that copious and frequent instillations were made for an hour.

"In a recent communication to me concerning gleditschine, from one of the chemists who have been investigating the matter, it was said that Dr. Rusby had stated to them that the natives of Bolivia, whenever they are unable to obtain supplies of coca leaves, chew the leaves of a tree which is closely allied to the gleditschia, and which leaves they call *chinci-coca.*

"Those who, it is alleged, have made this discovery have been denounced in the most unmeasured .erms by certain chemists. I is, therefore, in the highest degree proper that the plain and un varnished truth should be known. It is to be hoped that we ar approaching the finale of this most interesting and perplexing que tion.

The confounding of the names "gleditschine" and "stenocarpine" arose in this way: Goodman described the tree as a thorny growth, stating that its local name in Louisiana was "Tear Blanket." Dr. Seward, a chemist of Bergen's Point, N. J., thereupon declared it to be the *acacia stenocarpa*; hence, "steno carpine;" but Dr. Claiborne has investigated the subject thoroughly, and there can be no doubt that the leaves from which Goodman claims to have first made the extract were those of the Honey Locust, the *Gleditschina Triacanthos* of Linneus, named in honor of J. G. Gleditsch, "a contemporary botanist of Louisiana."

The *Philadelphia Medical and Surgical Reporter* of Nov. 5, at the close of an article on this subject, has a "note of caution,',in which the author, Dr. Turnbull, hints at the charge of counterfeiting, of which we have spoken, in the following language, "another cause of hesitation has been the counterfeiting of certain labels of eminent chemists products," but calls no names. Evidently, Lehn & Fink are fairly caught out by Parke Davis & Co., who deserve the thanks of the profession for this timely exposure of a contemptible attempt at fraud, whereby honorable physicians were for awhile placed in a compromising position. States prison was made for such "enterprising chemists" as those who would attempt to palm off a solution of cocaine for a newly discovered anæsthetic; it was clearly an attempt at fraud on the part of some one, or combination—they no doubt anticipated a heavy demand, and seemingly proposed to meet it with solution of cocaine at a big price; six dollars, they charged P. D. & Co. for an ounce of the alleged solution.

We await the result of the proposed test, with much interest; and as the distinguished editors of the two leading N. Y. weekly medical journals have agreed to supervise the test and report the result, the profession may rest assured that very shortly they will have the *truth*, the bottom facts, in this vexed question.

Dr. Claiborne, guided by Dr. S. P. Walker, of N. Y., an Alabamian, recognized the Honey Locust in a clump of trees growing in Central Park, N. Y.

