# Errata

**Published:** 1887-11

**Authors:** 


ERRATA. When it was too late to correct it, we found the intelligent compositor had made us say "indigenious" for "indigenous," in fourth line of leading editorial. For fear some hypercritical contemporary will pounce upon it, we point it out here, and now. "Excuse haste and a bad pen." No doubt there are other errors, overlooked; if so, we will have to take the criticism and the consequences. It is said Homer once nodded, and even that there are spots on the sun.

